# Quantitative Description of Surface Complementarity of Antibody-Antigen Interfaces

**DOI:** 10.3389/fmolb.2021.749784

**Published:** 2021-09-30

**Authors:** Lorenzo Di Rienzo, Edoardo Milanetti, Giancarlo Ruocco, Rosalba Lepore

**Affiliations:** ^1^ Center for Life Nano and Neuro-Science, Istituto Italiano di Tecnologia, Rome, Italy; ^2^ Department of Physics, Sapienza University, Rome, Italy; ^3^ Department of Biomedicine, Basel University Hospital and University of Basel, Basel, Switzerland

**Keywords:** surface complementarity, antibody complementarity determining regions, antibody—antigen complex, antigen recognition, zernike polynomials

## Abstract

Antibodies have the remarkable ability to recognise their cognate antigens with extraordinary affinity and specificity. Discerning the rules that define antibody-antigen recognition is a fundamental step in the rational design and engineering of functional antibodies with desired properties. In this study we apply the 3D Zernike formalism to the analysis of the surface properties of the antibody complementary determining regions (CDRs). Our results show that shape and electrostatic 3DZD descriptors of the surface of the CDRs are predictive of antigen specificity, with classification accuracy of 81% and area under the receiver operating characteristic curve (AUC) of 0.85. Additionally, while in terms of surface size, solvent accessibility and amino acid composition, antibody epitopes are typically not distinguishable from non-epitope, solvent-exposed regions of the antigen, the 3DZD descriptors detect significantly higher surface complementarity to the paratope, and are able to predict correct paratope-epitope interaction with an AUC = 0.75.

## 1 Introduction

Antibodies, also known as immunoglobulins, are multimeric Y-shaped proteins that the immune system uses to recognize and neutralize foreign targets, named antigens. The antigen binding site is located on the upper tip of the molecule, and is formed by the pairing of two variable domains, the VH and the VL, each contributing three hypervariable loops or complementary determining regions (CDR). The remarkable ability of the antibodies to recognize virtually any foreign antigen stems from the sequence and length variability of the CDR, while the framework of the molecule is largely conserved (Chothia and Lesk, 1987; Chothia et al., 1989; Tramontano et al., 1990).

Early studies, based on a handful of crystallographic structures, revealed that despite the large sequence variability of CDRs, five out of the six hypervariable loops only exhibit a limited number of main-chain conformations called “canonical structures” (Chothia and Lesk, 1987; Chothia et al., 1989), where most sequence variations only modify the surface generated by the side chains on a canonical main-chain structure. Over the years, with more experimentally determined structures of antibodies becoming available, an exhaustive repertoire of canonical structures has been compiled and their relationship with the chain isotypes (Tramontano et al., 1990; Chothia et al., 1992; Foote and Winter, 1992; Tomlinson et al., 1995; Martin and Thornton, 1996; Chothia et al., 1998; Decanniere et al., 2000; Vargas-Madrazo and Paz-García, 2002; Chailyan et al., 2011; North et al., 2011; Kuroda and Gray, 2016) and packing mode of the antibody was extensively analysed (Chothia et al., 1985; De Wildt et al., 1999; Abhinandan and Martin, 2010; Jayaram et al., 2012; Dunbar et al., 2013a). This led to the development of fully automated pipelines for the prediction of immunoglobulin structures given their amino acid sequences, with predictions reaching near-native accuracy both at the global and local CDR level (Whitelegg and Rees, 2000; Marcatili et al., 2014; Messih et al., 2014; Dunbar et al., 2016; Lepore et al., 2017; Weitzner et al., 2017). In parallel, a major focus has been in understanding the structural and molecular basis of antibody function and, in particular, of antigen recognition. The identification of the portion of the antigen that is recognized by an antibody, i.e. the epitope, is indeed of central relevance for the development of vaccines and immunodiagnostics, as well as for our understanding of protective immunity (Pollard and Bijker, 2020). As a consequence, in the past years, there have been several attempts in the direction of relating the sequence and structural properties of antibody binding sites to their function, and more specifically, to the type of recognised antigen. Early work by Webster et al. in 1994 first discovered a strong correlation between the topography of the CDRs and the broad nature of the antigen, proposing that antibodies binding protein antigens are characterised by flat combining sites, while those recognising smaller antigens, like haptens and peptides, show the most concave interfaces (Webster et al., 1994). Subsequent work confirmed and extended these findings to the length and sequence composition of the CDRs based on increased availability of sequence and structural data of antibody-antigen complexes (MacCallum et al., 1996; Collis et al., 2003; Lee et al., 2006; Raghunathan et al., 2012).

The study of molecular interactions in proteins, and antibodies in particular, poses well known challenges. Existing experimental methods, such as Xray crystallography, mass spectrometry, phage display and mutagenesis analysis are intrinsically expensive, laborious, and time consuming (Sela-Culang et al., 2013). Hence, computational methods have established themselves as a valuable complement to experimental biology efforts for the analysis and characterization of the vast repertoire of molecular interactions at the atomic level. Early studies by Lee and Richards (1971) proposed the first description of protein solvent-accessible surface, which was later refined by Connolly (1983), allowing to distinguish surface atoms from buried atoms and opening the way to efficient graphical representation and comparison of molecular surface properties. Subsequent methods relied on the application of spherical harmonics descriptors (Leicester et al., 1988; Max and Getzoff, 1988) and Fourier correlation theory to shape complementarity and electrostatic interaction analysis (Gabb et al., 1997). Additionally, approaches based on tessellation (Walls and Sternberg, 1992; Li et al., 2007), void volume (Jones and Thornton, 1996) and surface density (Norel et al., 1995) provided an efficient way for representation and matching of protein surfaces, including protein-protein interaction sites, ligand binding sites and functional sites (Via et al., 2000; Mitra and Pal, 2010).

In this study we rely on a surface representation of antibodies and their cognate antigens based on the 3D Zernike Descriptors (3DZD). The Zernike polynomials were first described by Fritz Zernike in 1934 (Zernike and Stratton, 1934) as a framework for the analysis of aberrations in optical systems and subsequently generalized to three-dimensions (Ming-Kuei Hu, 1962; Canterakis, 1999; Novotni and Klein, 2004). One of the convenient features of Zernike polynomials is that their rotational symmetry allows the polynomials to be expressed as products of radial terms and functions of angle, where the coordinate system can be rotated without changing the form of the polynomial. Hence, they allow a concise, roto-translationally invariant characterization of 3D objects, comparing favourably to other moment-based descriptors in terms of shape retrieval and robustness to topological and geometrical artifacts (Novotni and Klein, 2004). When applied to molecular surfaces, the 3DZD have been shown to capture both global and local protein surface properties and to adequately represent their physico-chemical properties (Venkatraman et al., 2009a; Venkatraman et al., 2009b; Kihara et al., 2011; Di Rienzo et al., 2017; Daberdaku and Ferrari, 2018; Daberdaku and Ferrari, 2019; Alba et al., 2020; Di Rienzo et al., 2020). Here we apply the 3DZD to provide a quantitative description of the shape and electrostatic properties of Ab–Ag interfaces, leading to an accurate classification of the antibodies according to the type of their cognate antigens solely based on the information of the CDR surface, with overall AUC = 0.85 and accuracy of 81%.

Additionally, we show that while in terms of surface size, solvent accessibility and amino acid composition, antibody epitopes are not distinguishable from non-epitope, solvent-exposed regions of the antigen, they display significantly higher surface complementarity to the antibody paratope, both in terms of shape and electrostatic 3DZD, leading to a prediction performance in terms of ROC AUC of 0.75 and 0.61 respectively.

## 2 Materials and Methods

### 2.1 Dataset

We selected 326 antibodies with redundancy lower than 90% and resolution <3.0 Å using the SabDab database (Dunbar et al., 2013b). 229 antibodies were solved in complex with protein antigens, 71 with haptens, 19 with carbohydrates and 7 with nucleic acids. The sequence of each antibody was renumbered according to the Chothia numbering scheme (Chothia and Lesk, 1987; Chothia et al., 1989) using an in-house python script.

### 2.2 Solvent Accessible Surface and Electrostatics Surface

For each antibody and protein antigen 3D structure, atomic partial charges and radii were assigned using PDB2PQR with default parameters (Dolinsky et al., 2004). Solvent Accessible Surface (SAS) was computed using GROMACS (Abraham et al., 2015). Electrostatic surface (ES) potential was computed using the Bluues software (options -srf and -srfpot) (Fogolari et al., 2012). Each molecular surface point was assigned to the electrostatic potential of the corresponding residue. The “geometry” (Habel et al., 2019) and “Bio3D” (Grant et al., 2006) packages available in R were used for PDB structure processing and analysis.

### 2.3 Voxelization Procedure

The set of selected molecular surface points was scaled to the unit sphere and placed into a 3D grid of dimension 128^3^. To avoid boundary effects, the size of the bounding box of the point cloud was set so as to be contained within 80% of the unit sphere (Grandison et al., 2009). Voxelization was performed separately for SAS and ES. In SAS voxelization, each voxel was assigned a value of 1 if the center of the voxel was closer than 1.7 to any SAS point, 0 otherwise. In ES voxelization, each voxel was assigned the mean ES value of the enclosed points, 0 otherwise.

Since the Zernike formalism does not differentiate positive and negative values (Chikhi et al., 2010; Daberdaku and Ferrari, 2018), but only patterns of non-zero values in the 3D space, voxels were initialized for positive and negative patterns separately using a similar approach as done in (Chikhi et al., 2010), as follows:

$$f_{elec}^{+}=0\;\;\;\;if\;\;\;f_{elec}<0\;\;\;\;\;\;\;\;f_{elec}^{+}=f_{elec}\;\;\;if\;\;\;f_{elec}>0$$ (1)

$$f_{elec}^{-}=f_{elec}\;\;\;\;if\;\;\;f_{elec}<0\;\;\;\;\;\;\;\;f_{elec}^{-}=0\;\;\;if\;\;\;f_{elec}>0$$ (2)

In summary, voxels with positive electrostatics values were initialized to 1 and all other voxels with negative electrostatics values were set to zero, and vice versa. The resulting voxels, one for SAS values, and two for positive and negative ES values, respectively, were considered as three different 3D functions, f(x), each expanded into the 3DZD as described in the next section.

### 2.4 3D Zernike Descriptors

For the quantitative description of the binding sites, we rely on a representation based on the Zernike polynomials and their corresponding moments. Moment-based representations are a class of mathematical descriptors of shape, originally developed for pattern recognition and subsequently generalized to three-dimensions (Ming-Kuei Hu, 1962; Canterakis, 1999; Novotni and Klein, 2004).

A surface described by a function *f* (*r*, *θ*, *ϕ*) in polar coordinates can be represented by a series expansion in an orthonormal sequence of polynomials (Canterakis, 1999):

$$f\left(r,\theta ,\phi \right)=\overset{\infty }{\underset{n=0}{\sum }}\overset{n}{\underset{l=0}{\sum }}\overset{l}{\underset{m=-l}{\sum }}C_{nlm}Z_{nl}^{m}\left(r,\theta ,\phi \right)$$ (3)

where the indices n, m and l are the order, degree and repetition, respectively.

The Zernike polynomials can be written as:

$$Z_{nl}^{m}\left(r,\theta ,\phi \right)=R_{nl}\left(r\right)Y_{l}^{m}\left(\theta ,\phi \right)$$ (4)

where the Y functions are complex spherical harmonics depending on both *θ* and *ϕ* while R only depends on the radius r, which is given by

$$R_{nl}\left(r\right)=\overset{\frac{\left(n-l\right)}{2}}{\underset{k=0}{\sum }}N_{nlk}r^{n-2k}$$ (5)

where N is a normalization factor.

The 3D Zernike moments of a surface described by a function *f* (*r*, *θ*, *ϕ*) are defined as the coefficients of the expansion of f(r) in the Zernike polynomial basis, i.e.:

$$C_{nlm}=\int_{|r|\leq 1}f\left(r\right)\bar{Z_{nl}^{m}\left(r,\theta ,\phi \right)}dr$$ (6)

where $\bar{Z}$ is the polynomial complex conjugate.

Their rotation invariant norms, i.e. the 3DZD, are defined as:

$$D_{nl}=\|C_{nlm}\|=\sqrt{\overset{l}{\underset{m=-l}{\sum }}{\left(C_{nlm}\right)}^{2}}.$$ (7)

The Zernike formalism can be as detailed as desired by modulating the order of the expansion n. In our implementation, the function f represents the geometric or the (positive or negative) electrostatic potential of the molecular surface, and the maximum order of expansion was set to 20, giving a total of 121 invariants.

### 2.5 Generation of Native Epitopes and Surface Decoys

Given the dataset of Antibody-Antigen complexes containing protein antigens, the native geometric epitope was defined as the set of residues of the antigen having a distance lower than 6 Å to any residue of the antibody. The pivot residue was defined as the residue with the lowest mean distance to any residue of the native geometric epitope. The native electrostatic epitope was defined as the set of residues of the antigen having a distance shorter than 15 Å to any residue of the antibody. For the set of native geometric epitope residues, the Solvent Accessible Surface Area (SASA) was computed using GROMACS. The mean and standard deviation values of the computed global and residue-based SASA were used to generate an alternative set of surface patches, i.e. decoy epitopes. The algorithm first selects a decoy pivot residue, i.e. by randomly selecting any solvent exposed residue having a value of SASA within half standard deviation of the mean SASA value measured over all pivot residues of the native epitopes, i.e. *SASA* = 0.48 ± 0.33 nm^2^ (Supplementary Figure S1). The algorithm proceeds by adding neighboring solvent accessible residues, i.e. having relative SASA >0.2 (Tien et al., 2013), until the decoy geometric epitope reaches a similar global SASA to that of the native epitope. To ensures continuous coverage of the antigen protein surface (Supplementary Figure S2) and diversity of the generated patches, a maximum 50% surface patch overlap was allowed between native and decoy epitopes. Electrostatic decoy epitopes were defined by calculating the electrostatic potential over the region defined by a geometric decoy epitope considering all the charged residues within 15 $\overset{{}^{\circ}}{A}$ to the pivot residue.

### 2.6 Comparison of the 3DZD Descriptors

Given a pair of ordered set of 3DZD, x and y, their cosine distance is measured as:

$$D\left(x,y\right)=1-S_{c}\left(x,y\right)=1-\frac{xy}{\left\|x\|\;\|y\right\|}$$ (8)

where *S*_*c*_ (*x*, *y*) is the cosine similarity as measured by the “proxy” R package (Meyer and Buchta, 2019).

Given two patches A and B, the similarity between their 3DZD is computed as:

$${\left[A-B\right]}_{shape}=D\left(X_{shape}^{A},X_{shape}^{B}\right)$$ (9)

$${\left[A-B\right]}_{elec}=\frac{\left(D\left(X_{elec}^{+,A},X_{elec}^{+,B}\right)+D\left(X_{elec}^{-,A},X_{elec}^{-,B}\right)\right)}{2}$$ (10)

where *X*_*shape*_, $X_{elec}^{+}$ and $X_{elec}^{-}$ are, respectively, the shape, the electrostatic positive potential, and the electrostatic negative potential 3DZD.

The surface complementarity between A and B is defined as follows:

$${\left[A-B\right]}_{shape}=D\left(X_{shape}^{A},X_{shape}^{B}\right)$$ (11)

$${\left[A-B\right]}_{elec}=\frac{\left(D\left(X_{elec}^{+,A},X_{elec}^{-,B}\right)+D\left(X_{elec}^{-,A},X_{elec}^{+,B}\right)\right)}{2}$$ (12)

## 3 Results

In this work we aim at providing a quantitative description of the geometric and electrostatic properties of antibody-antigen interaction through a mathematical representation of the interacting surfaces. To this aim, we rely on a dataset of experimentally determined 3D structures of antibody-antigen complexes and a moment-based representation of the interacting surface using the 3D Zernike descriptors (3DZD) (Novotni and Klein, 2004; Venkatraman et al., 2009b; Daberdaku and Ferrari, 2018).

**FIGURE 1:**
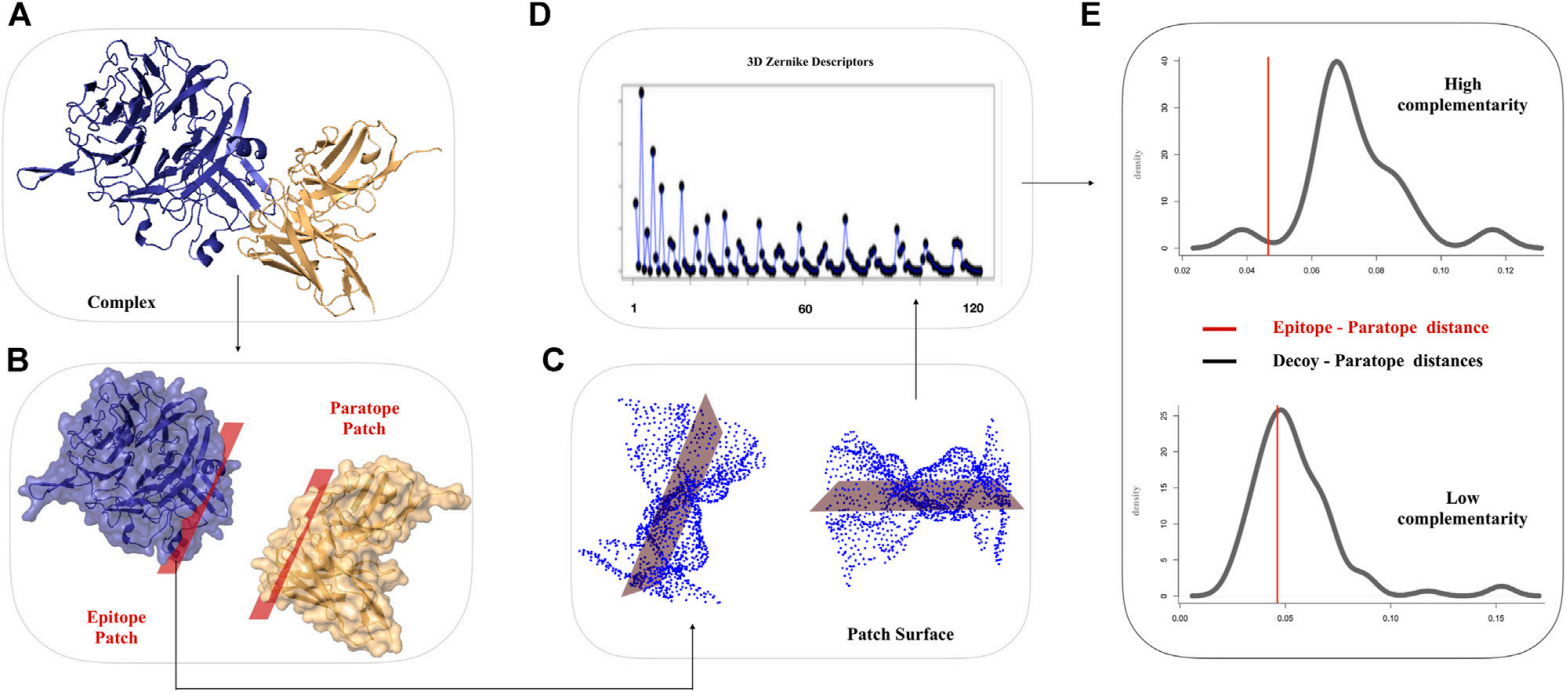
Schematic workflow for the comparison of Ab-Ag interfaces based on 3DZD. **(A)** Molecular representation of a given Ab-Ag complex. Antibody and antigen are shown in gold and blue, respectively. **(B)** The interacting surfaces are selected according to inter-molecular atomic distance threshold. **(C)** Solvent accessible and electrostatic surfaces are computed on the selected regions **(D)** 3DZD Zernike descriptors are computed for each molecular surface. **(E)** Distribution of 3DZD surface complementary complementarity between paratope and non-epitope surface decoys. The red line denotes 3DZD surface complementarity between the antibody paratope and their cognate epitope.

The 3DZD descriptors provide a compact, roto-translationally invariant representation of 3D objects, thus enabling effective comparison of both global and local properties of molecular surfaces by standard pairwise similarity metrics. The order n of the series expansion determines the resolution of the descriptor. In this study, 3DZD were computed at different levels of truncation of the expansion, with n ranging from 10 to 20, which correspond to vectors of 36 and 121 invariants, respectively. The overall scheme of the procedure used in this work is shown in Figure 1.

### 3.1 Antibody Classification Based on Surface Shape and Electrostatic 3DZD Descriptors of CDRs

**FIGURE 2:**
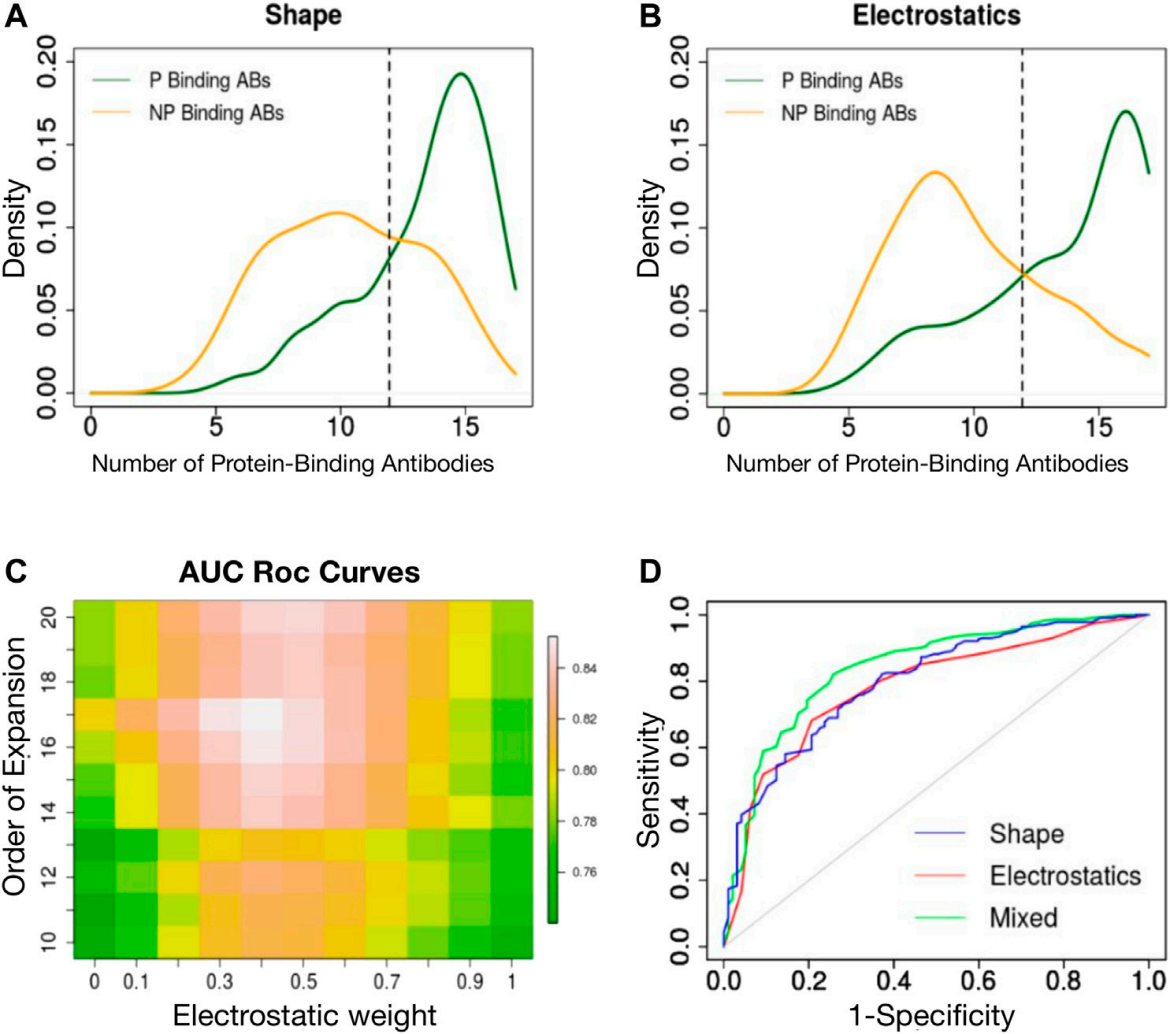
**(A)** Density distribution of protein binding antibodies (*N*_*pb*_) in the neighbours set of protein binding (green curve) and non-protein binding antibodies (orange curve) based on surface shape similarity. **(B)** Density distribution of protein binding antibodies (*N*_*pb*_) in the neighbours set of protein binding (green curve) and non-protein binding antibodies (orange curve) based on electrostatic surface similarity. **(C)** Classification performance (ROC AUC) is reported as a function of the order n of the Zernike expansion and weight of the average. **(D)** ROC curve of the best classifier based on shape 3DZD (blue curve), electrostatic 3DZD (red curve) and weighted average *N*_*pb*_ (green curve).

We have previously shown that a 3DZD-based description of the surface of the antibody CDRs provides an effective metric for antibody classification according to their specificity towards protein and non-protein antigens (Di Rienzo et al., 2017). Here we extend this approach to the analysis of both the shape and electrostatic properties of the CDRs and analyze the classification performance of both descriptors at different orders of the Zernike expansion. For each CDR we generated two sets of 121-dimensional vectors, representing the 3DZD of the shape and the electrostatic surface, similar to what done in (Chikhi et al., 2010; Di Rienzo et al., 2020). The similarity between each set of descriptors is then computed to perform an all-against-all comparison of CDRs, according to Eq. 9, 10 in Methods section. For each CDR, we then selected the nearest neighbors set as the 5% most similar CDRs in terms of shape and electrostatic surface and analyse the number of protein binding antibodies (*N*_*pb*_) in the neighbours set. As it is shown in Figures 2A,B, protein-binding antibodies (green curve) are typically characterized by an higher number of *N*_*pb*_ ( $mean\left(N_{pb}^{shape}\right)=13.37\pm 2.61$ , $mean\left(N_{pb}^{elec}\right)=13.54\pm 3.24$ ) in the neighbors set as compared to non protein-binding antibodies (orange curve) ( $mean\left(N_{pb}^{shape}\right)=10.31\pm 2.99$ , $mean\left(N_{pb}^{elec}\right)=9.93\pm 3.13$ ) and to random expectation (i.e., *Ex* [*N*_*pb*_] = *N*_*Prot*_/*N*_*tot*_, where *Ex* [*N*_*pb*_] is the expected number of protein-binding antibodies if they were distributed uniformly, *N*_*prot*_ represents the number of protein-binding in the dataset and *N*_*tot*_ is the total number of antibodies in the dataset.).

We next analyzed the performance of each descriptor in classifying the CDRs as a function of the antigen type, using a leave-one-out approach. In summary, for each CDR, if the *N*_*pb*_ was greater than Ex (*N*_*pb*_) the CDR was labeled as protein-binding, non protein-binding otherwise. The obtained classification accuracy for the shape and electrostatic descriptors at order *n* = 20 is 75 and 73%, respectively. Using a Receiver Operating Curve (ROC) analysis, both descriptors achieved an Area Under the Curve (AUC) of 0.78. We next analyzed the classification performance when assigning the class label based on the weighted contribution of shape and electrostatics, as follows:

$$\bar{N_{pb}}=AN_{pb}^{elec}+\left(1-A\right)N_{pb}^{shape}\;\;\;A\in \left[0,1\right]$$ (13)

where $N_{pb}^{shape}$ and $N_{pb}^{elec}$ correspond to the *N*_*pb*_ computed based on shape and electrostatic descriptors, respectively, and A is the weight ranging from 0 to 1. The results are shown in Figure 2C, where the ROC AUC is reported as a function of the weight A and the order n of the Zernike expansion. As it can be noticed, overall performance increases with increasing values of n. Higher AUC values are achieved when both descriptors contribute with similar weight in the classification. Top classification performance indeed is obtained with A = 0.4 and *n* = 17, leading to an AUC = 0.85 and accuracy of 81%. A very similar performance is obtained with *n* = 20 and A = 0.4 (AUC = 0.83).

### 3.2 CDRs vs. Antibody Paratope

The sequence and structure analysis of antibodies, as well as antibody engineering experiments, crucially rely on the precise identification of the CDRs from the antibody sequence (Chothia and Lesk, 1987; Chothia et al., 1989; Kabat et al., 1992; MacCallum et al., 1996; Lefranc, 2011). On the other hand, it is well known that the CDRs only provide a proxy of the actual antigen-binding site, i.e. the antibody paratope (Kunik et al., 2012; Olimpieri et al., 2013). Indeed, early studies showed that only 20–30% of residues within the CDRs are directly involved in the interaction with the antigen (Padlan, 1994; Sela-Culang et al., 2013). To quantify to what extent this approximation affects our predictions, we analyzed the classification performance as a function of distance from the center of the antibody-antigen interface. For each Antibody-Antigen complex, we defined a *centerpoint*, *b*, as the centroid of the 10 interface atoms of the antibody closer to the antigen and computed the 3DZD for increasing concentric shells around *b*.

**FIGURE 3:**
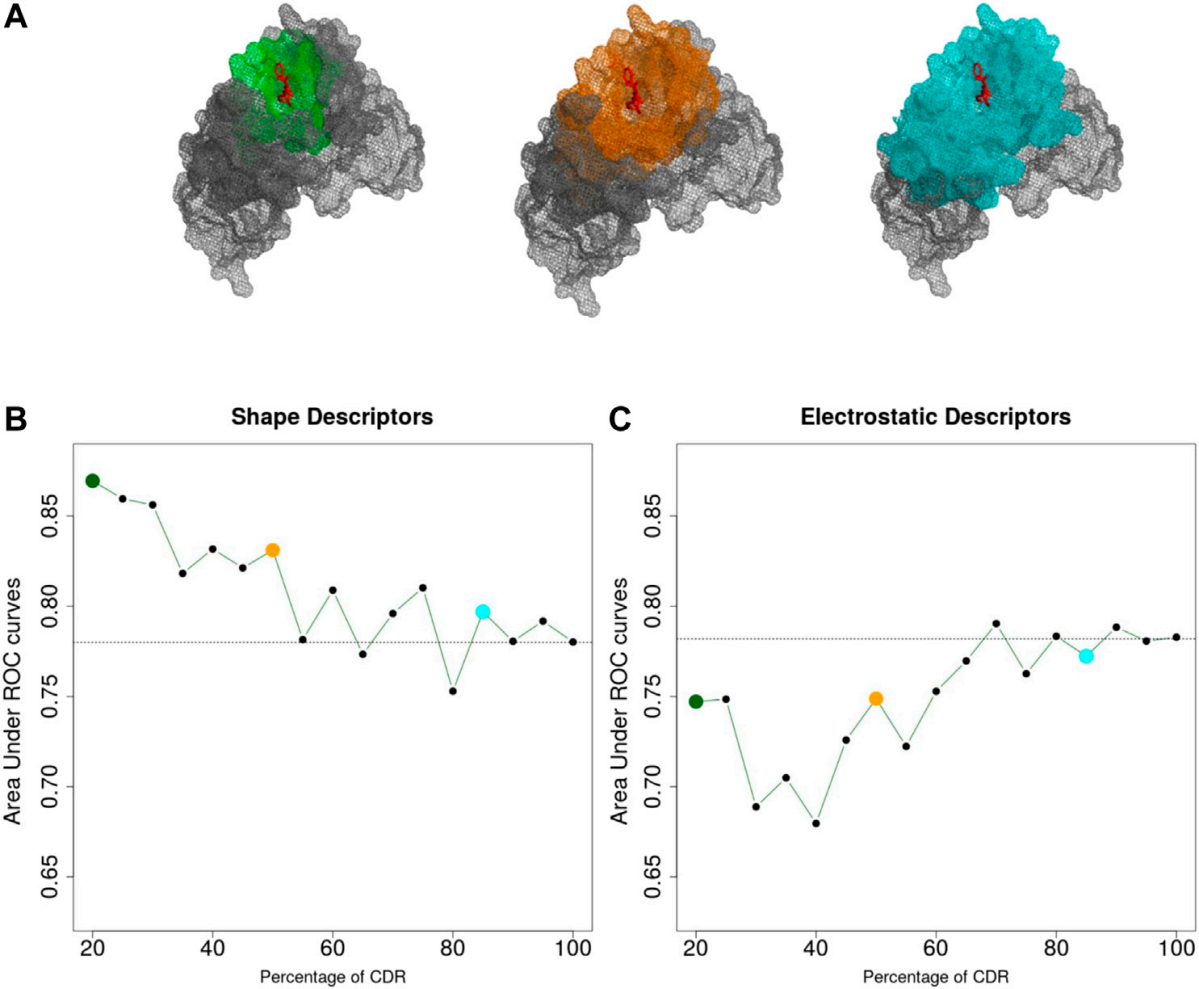
**(A)** Portion of the CDR surface used for classification. **(B,C)** Area Under the ROC Curve achieved considering different portions of the CDR, based on shape **(B)** and electrostatics **(C)** 3DZD descriptors. Dashed lines indicate the performances obtained considering the entire CDR surface (AUC = 0.78 for both descriptors).

We then applied the same classification procedure as described previously, by fixing the order *n* = 20 for both shape and electrostatic 3DZD. The results are shown in Figure 3 where the ROC AUC of the individual classifiers are reported as a function of the percentage of the CDR surface included in the analysis.

As it can be noticed in Figure 3B, the performance of the shape-based classifier shows a maximum when the selected surface region around b extends up to including 20% of the CDRs (ROC AUC = 0.88) followed by a linear decrease when larger surfaces are considered. These results are consistent with the previous notion that shape recognition of the antigen is largely mediated by smaller interacting surfaces contained within the CDR, i.e. the antibody paratope. In summary, while the overall CDR surface can inform about the function of the antibody, this analysis highlights that the information of the paratope can significantly increase our ability to predict antibody specificity. On the other hand, in Figure 3C, the classification performance based on the electrostatic descriptor shows a different trend. Indeed, while the classifier shows an overall lower performance compared to the shape-based classifier, performance increases when larger CDR surfaces are considered, reaching a maximum when almost the entire CDR surface is included in the analysis.

### 3.3 Geometric and Electrostatic Complementarity of Antibody-Antigen Interfaces

**FIGURE 4:**
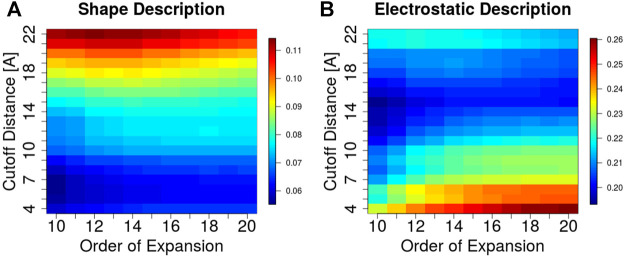
Surface complementarity of antibody-antigen interacting surfaces based on shape **(A)** and electrostatic **(B)** 3DZD descriptors as a function of the interaction cutoff distance (y-axis) and order n of the series expansion (x-axis).

**FIGURE 5:**
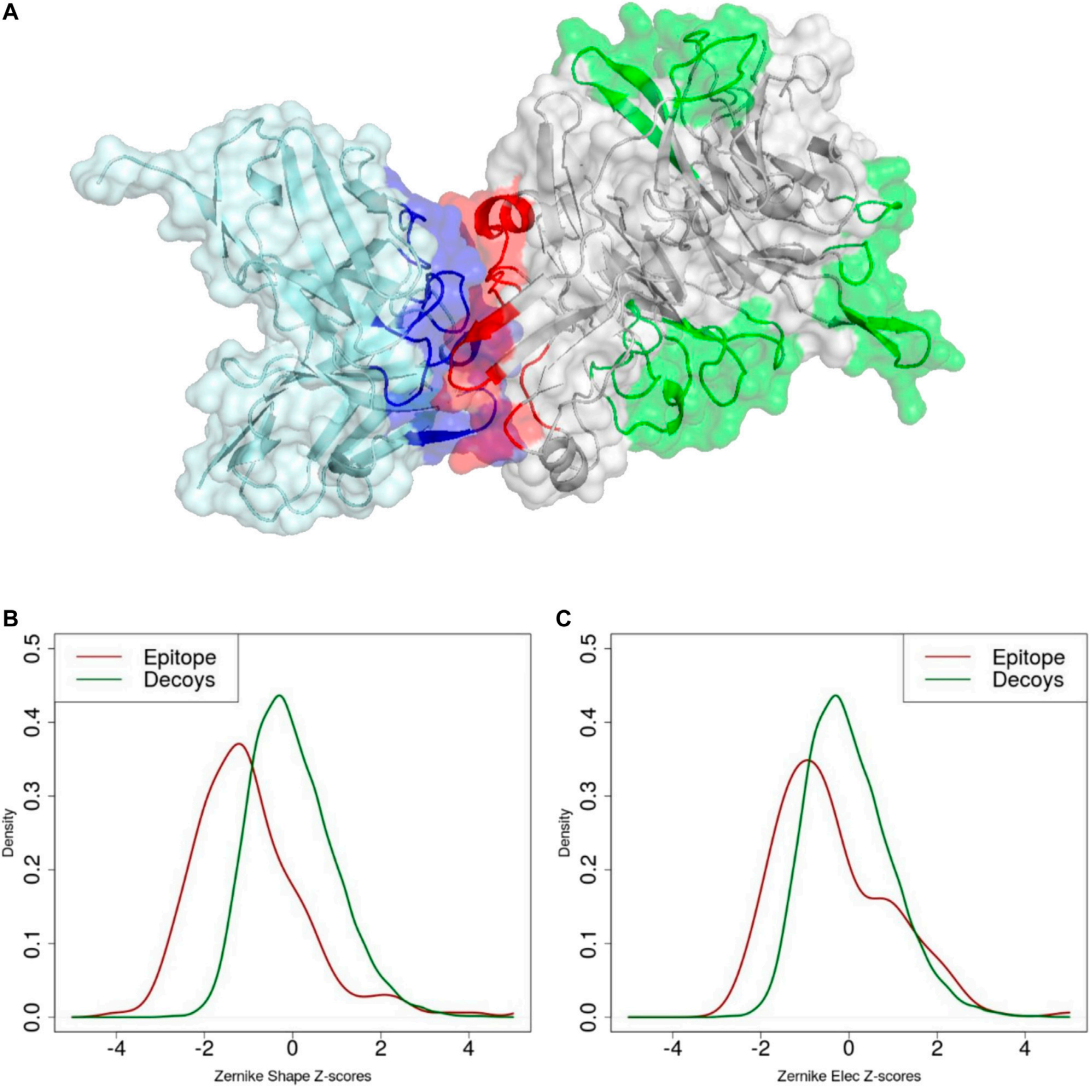
**(A)** Molecular representation of experimental paratope (blue), experimental epitope (red) and decoys (green). Z-score distribution of **(B)** shape and **(C)** electrostatic surface complementarity based on the 3DZD descriptors between paratope-epitope (red) and paratope-decoy surfaces (green).

A key feature of the 3DZD description is that it is invariant under rotation and translation of the represented surface. This implies that two interacting protein regions with perfect surface complementarity yield identical sets of 3DZD descriptors (Venkatraman et al., 2009a). In line with this principle, here we focus on the application of 3DZD to the analysis of surface complementarity between antibody CDRs and their cognate protein antigens (Details in Methods). The results are shown in Figure 4, where the average surface shape and electrostatic complementarity computed on 229 antibody-antigen complexes are reported as a function of the interaction cutoff distance between the antibody and the antigen, and the order n of the series expansion. As expected, shape complementarity decreases at higher values of the cutoff distance, i.e. as regions of the antibody/antigen that are distant from the interaction interface are progressively included in the analysis. On the other hand, electrostatic complementarity increases at higher distances, reaching a maximum when the distance cutoff is $\;15\overset{{}^{\circ}}{A}$ . Notably, in both cases, results are consistent at different orders n of the series expansion. These results indicate that the two descriptors are competent in capturing both short- and long-range effects occurring during antibody-antigen recognition. As further validation of our approach, we measured the surface complementarity at the paratope-epitope interface and compared it with that measured between the paratope and a set of non-epitope, solvent-exposed regions of the antigen, i.e. surface decoys. The results are reported in Figure 5, where both shape and electrostatic complementarity are reported for each paratope as normalized Z-score distances to native epitopes and surface decoys, respectively. Notably, while in terms of amino acid composition, surface size, and solvent accessibility the antibody epitopes are essentially not distinguishable from the decoys (Supplementary Figure S3), they display significantly higher surface shape and electrostatics complementarity to the paratope. In summary, the metric is able to distinguish the correct paratope-epitope pair among the set of decoys with a classification performance of AUC = 0.75 based on the shape descriptor, and AUC = 0.61 based on the electrostatic 3DZD. Additionally, we compared the 3DZD complementarity observed between specific paratope-epitope pairs and that between the antibody paratopes and non-native epitopes. The results (Supplementary Figure S4) show that only a relatively low number, i.e. 68% (72%) of the antibodies in our dataset show a higher shape (electrostatic) complementarity to their cognate epitope compared to non-native epitopes, highlighting the limitation of this metric in the very elusive task of predicting which antibody recognises specifically a given antigen.

## 4 Discussions

In this work we describe a computational protocol based on the 3D Zernike descriptors formalism, which allows a fast, superposition-free comparison of molecular surfaces, and has been applied here to the study of the interacting regions of the antibodies and their cognate antigens. The method represents a significant upgrade compared to our previous implementation (Di Rienzo et al., 2017) as it includes two relevant modifications found to improve its performance, namely, the selection of the molecular patch of interest and the description of its electrostatic properties. Using this new version of the method we are able to classify the antibodies according to the nature of their recognized antigens with a classification performance of 81%. Notably, the method only takes as input the information of the antibody CDR surface. However, when the analysis is restricted to the CDR surface that is in direct contact with the antigen, i.e. the antibody paratope, the classifier based on the shape 3DZD descriptor alone reaches a maximum performance of AUC = 0.88.

As 3DZD descriptors are roto-translation invariant, they are also adept at capturing and quantifying surface complementarity at protein-protein interfaces (Venkatraman et al., 2009a). Here we exploit this property to study the surface shape and electrostatic complementarity between antibody CDRs and their bound protein antigens. Our results indicate that maximum surface shape complementarity is achieved at the docking interface, i.e. at 4 to 8 Angstrom distance cutoff between antibody and antigen residues, and decreases when larger distance cutoffs are considered. In contrast, electrostatic complementarity increases at larger distance cutoffs, reaching a maximum between 14 and 17 Å. For both descriptors, results are consistent at different orders n of the series expansion. Hence, we tested the ability of the surface complementarity metric in recognising antigenic surface epitopes among a set of non-epitope, solvent exposed regions of the antigen, i.e. surface decoys. Notably, while in terms of surface size, solvent accessibility and amino acid composition the selected surface decoys are not distinguishable from true epitopes, they display significantly lower surface complementarity to the paratope. Indeed, when the 3DZD-based complementarity metric is used to select the correct paratope-epitope pair among a set of surface decoys, we show that shape complementarity alone can lead to a prediction performance of ROC AUC = 0.75. These results show that 3DZD provide a valid quantitative metric for the analysis of surface complementarity at the antibody-antigen interface, which is expected to find applications in many areas, including the identification and design of optimal antibody-antigen interfaces.

## Data Availability

The original contributions presented in the study are included in the article/Supplementary Material, further inquiries can be directed to the corresponding author.
