# Supplementary material for: Quantitative Description of Surface Complementarity of Antibody-Antigen Interfaces
Source: Front Mol Biosci. 2021 Sep 30;8:749784. doi: 10.3389/fmolb.2021.749784 (PMC8514621; doi:10.3389/fmolb.2021.749784)
Supplement: Supplementary file 1 [file DataSheet1.PDF]

Supplementary Information:
Quantitative description of surface complementarity of antibody-antigen interfaces

Lorenzo Di Rienzo,[1] Edoardo Milanetti,[2,1] Giancarlo Ruocco,[1,2] and Rosalba Lepore [13]

[1] *Center for Life Nano & Neuro-Science, Istituto Italiano di Tecnologia, Viale Regina Elena 291, 00161, Rome, Italy*

[2] *Department of Physics, Sapienza University, Piazzale Aldo Moro 5, 00185, Rome, Italy*

[3] *Department of Biomedicine, Basel University Hospital and University of Basel, Basel, Switzerland*

[1] Corresponding author: rosalba.lepore@unibas.ch

I. DISTRIBUTION OF SOLVENT ACCESSIBLE SURFACE AREA OF ANTIBODY EPITOPES

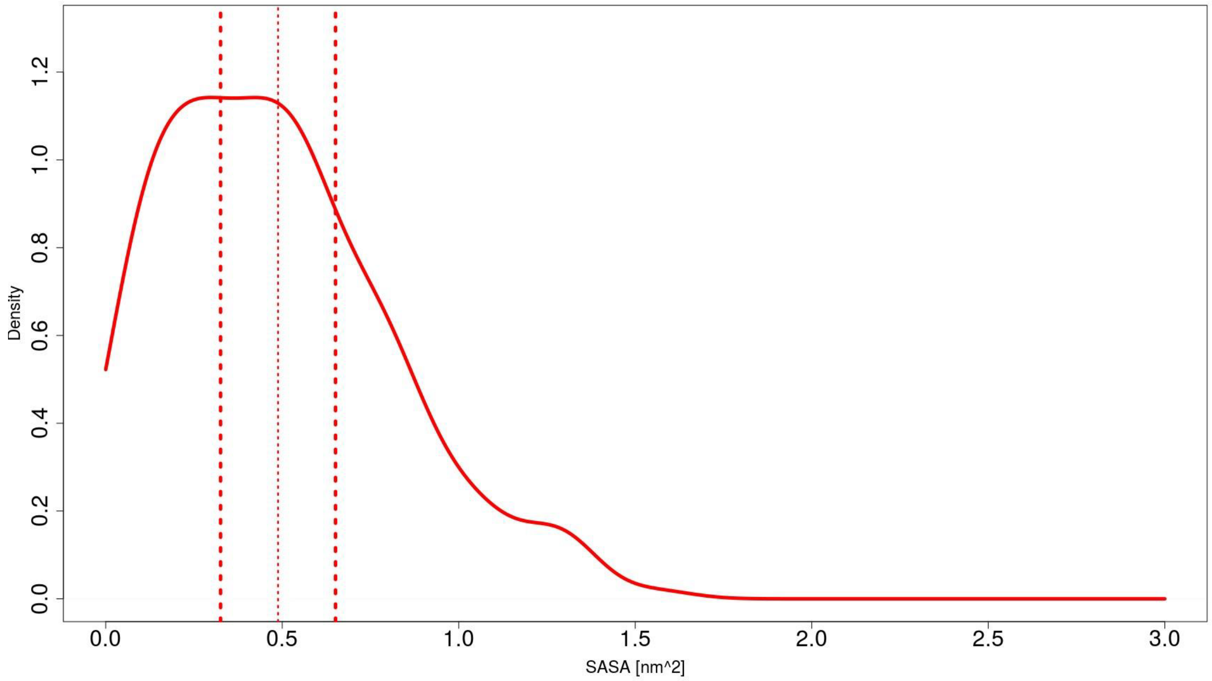

FIG. 1: The distribution of the Solvent Accessible Surface Area (SASA) for each epitope residue closer than 6 ˚A from the antibody (*the shape epitope*). Dashed lines denote the mean SASA (0.48 nm^2) +-1 standard deviation (0.33 nm^2 and 0.68 nm^2) computed over the central residues of the epitopes.

II. ANTIGEN SURFACE COVERAGE BY SURFACE DECOYS

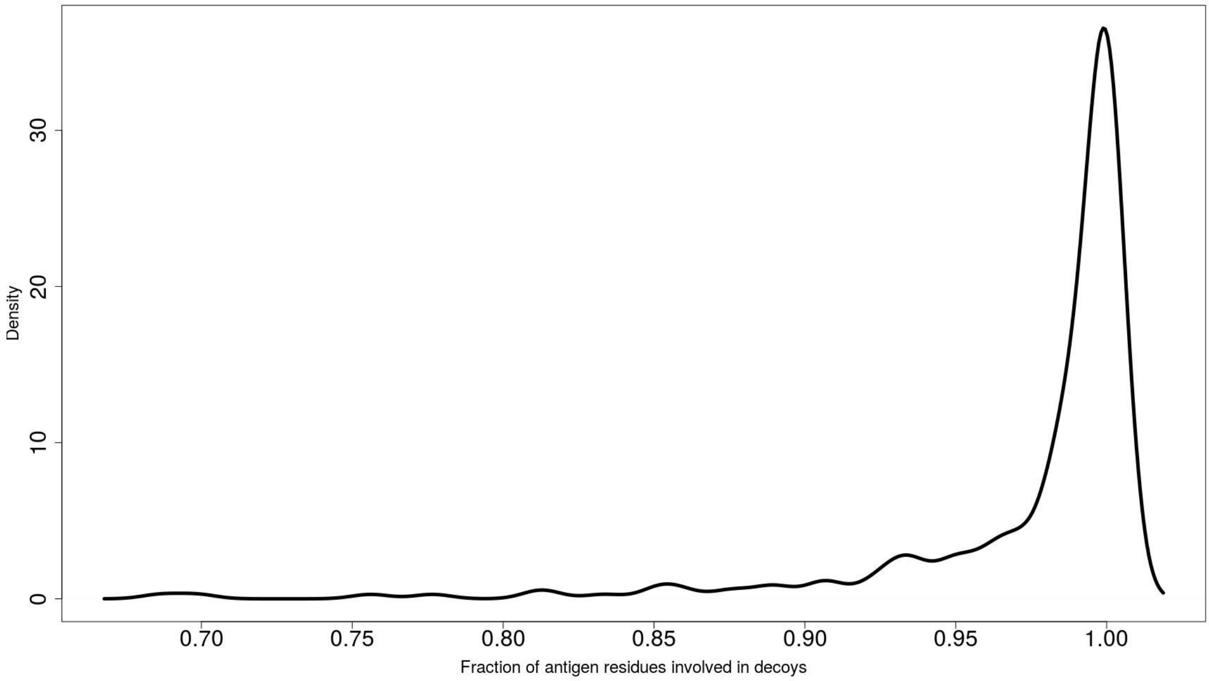

FIG. 2: Distribution of the fraction of antigen surface residues included in at least one surface decoy. Most antigens are sampled by at least 70% of their total surface area.

III. NATIVE EPITOPES VS SURFACE DECOYS

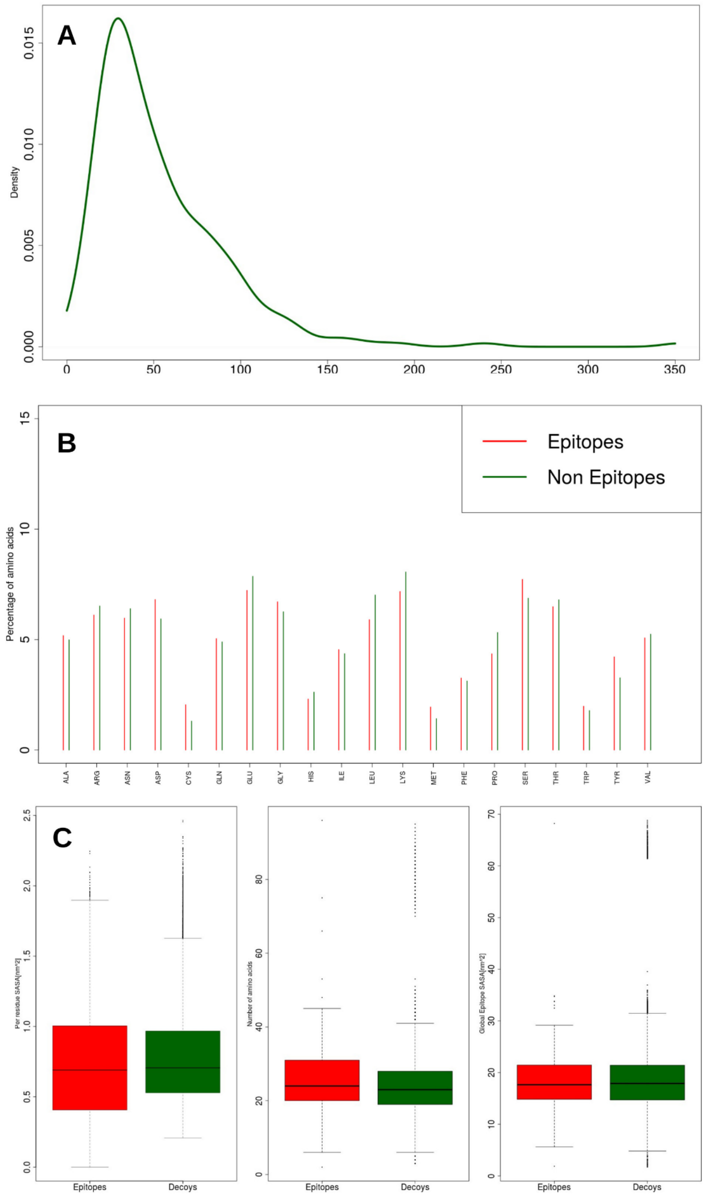

FIG. 3: The comparison between decoy and epitope properties. The green lines regard the decoys while the red lines represent the x-ray derived epitope. **A)** Distribution of the number of surface decoys generated for different antigens. The number of surface decoys varies with the size and total surface area of the protein antigen. **B)** Amino acid distribution observed in native epitopes (red lines) and surface decoys (green lines). **C)** Distribution of per-residue SASA, number of residues, and global SASA in native epitopes (red boxplots) and surface decoys (green boxplots).

IV. SPECIFIC AND NON-SPECIFIC COMPLEMENTARITY

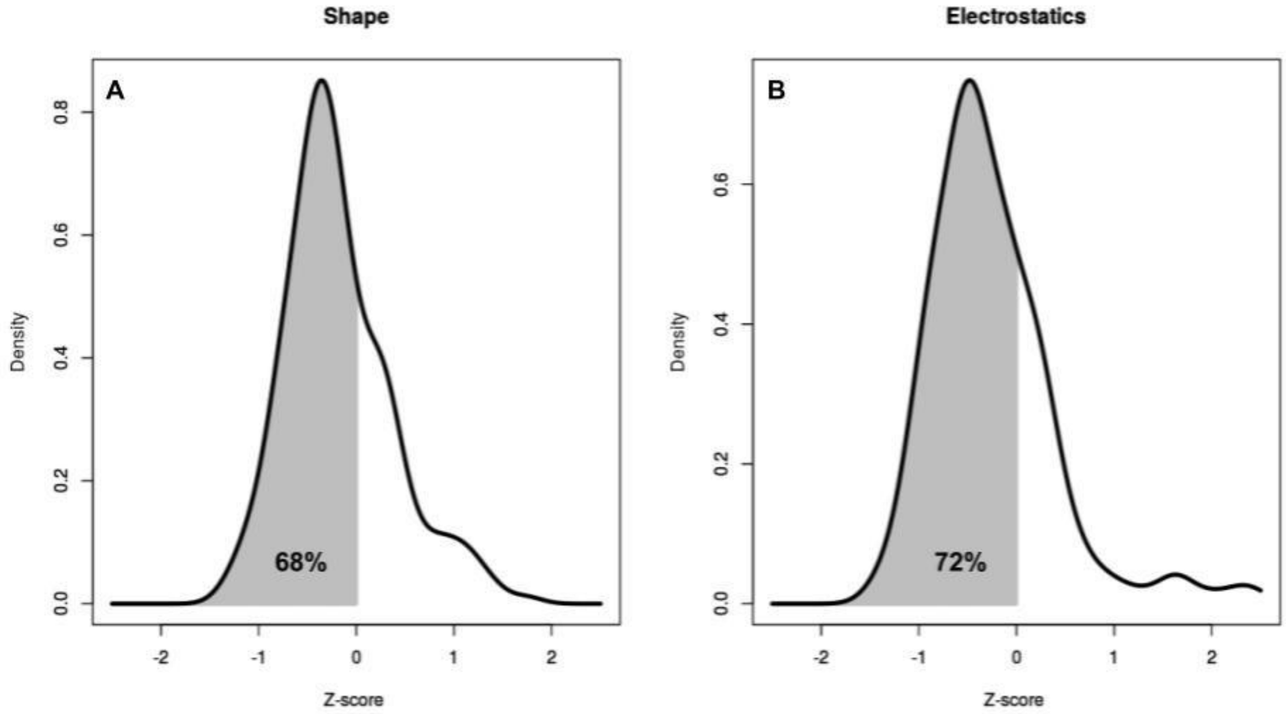

FIG. 4: Z-score distributions of the shape and electrostatic complementarity in specific and non-specific paratope-epitope pairs based on shape (**A**) and electrostatic (**B**) 3DZD descriptors. The shaded area represents the fraction of the antibody paratopes in the dataset showing higher complementarity to their epitope vs non-native epitopes.
